# *Drosophila melanogaster* as a rapid in vivo assay system for preclinical anti‐seizure medication testing

**DOI:** 10.1002/epi4.70101

**Published:** 2025-07-10

**Authors:** Emma V. Töfflinger, Konstantin L. Makridis, M.‐Marcel Heim, David Owald, Angela M. Kaindl

**Affiliations:** ^1^ Department of Pediatric Neurology Charité – Universitätsmedizin Berlin Berlin Germany; ^2^ Center for Chronically Sick Children, Charité – Universitätsmedizin Berlin Berlin Germany; ^3^ Charité – Universitätsmedizin Berlin, Institute of Cell‐ and Neurobiology Berlin Germany; ^4^ German Center for Child and Adolescent Health (DZKJ), Section CNS Development and Neurologic Disease, Partner Site Berlin Berlin Germany; ^5^ Institute of Neurophysiology, Charité – Universitätsmedizin Berlin Berlin Germany

**Keywords:** anti‐seizure medication, developmental and epileptic encephalopathy, Dravet syndrome, *Drosophila melanogaster*, epilepsy

## Abstract

Epilepsy represents a significant medical challenge, with a third of patients failing to achieve seizure freedom despite the use of multiple different anti‐seizure medications (ASM). Drug resistance is common in genetically caused epilepsies. Patients are faced with repeated, long‐lasting, and frequently frustrating drug testing, necessitating targeted therapies and drug repurposing. While in silico tools offer some insight, novel and often genetic epilepsies require preclinical models, which are expensive and time‐consuming. Here, we propose *Drosophila melanogaster* as a rapid in vivo model for preclinical ASM efficacy testing using the sodium channel‐associated epilepsies, Dravet syndrome (DS), and generalized epilepsy with febrile seizures plus (GEFS+) as model disorders. We utilize vinegar fly models of DS and GEFS+ that exhibit phenotypic similarities to human patients, including seizures and increased morbidity. Moreover, treatment with ASM effective in humans (clobazam, stiripentol, fenfluramine) reduces seizures, while the application of sodium channel blocking ASM (phenytoin) was deleterious, underlining the model's utility. The utilization of *Drosophila* as a preclinical model offers a promising avenue for studying genetic epilepsies and assessing ASM efficacy. This approach has the potential to facilitate the development of tailored treatments for patients using a rapidly available in vivo model.

**Plain Language Summary:**

Epilepsy is a challenging condition, with about one‐third of patients unable to control seizures despite trying multiple drug treatments. This is especially common in genetic epilepsies. Developing new treatments is expensive and slow, highlighting the need for faster, targeted approaches. This study uses the fly (*Drosophila melanogaster*) as a rapid, cost‐effective model to study genetic epilepsies like Dravet syndrome (DS). These fly models mimic key symptoms seen in humans, including seizures and shorter lifespans. Effective human anti‐seizure medications (e.g., clobazam, stiripentol, and fenfluramine) reduced seizures, while sodium channel blockers like phenytoin worsened them. The *Drosophila* model offers a promising and efficient way to study genetic epilepsies and test treatments, accelerating the development of more targeted therapies.

**Key points**

- Fruit fly models with *SCN1A* variants mimic Dravet syndrome and GEFS+ phenotypes, including heat‐induced seizures and reduced lifespan.
- Clobazam, stiripentol, and fenfluramine reduce seizures effectively; valproic acid has no significant effect.
- Phenytoin worsens seizures, mirroring its known contraindication in Dravet syndrome.
- *Drosophila melanogaster* preclinical ASM testing is fast, low‐cost, enables rapid genetic modeling, and avoids vertebrate‐specific ethics.
- *Drosophila melanogaster* enables preclinical evaluation of therapeutic efficacy and advancement of precision medicine in genetic epilepsies.

## 1 INTRODUCTION

Epilepsy is one of the most common neurological disorders worldwide. First‐line treatment with anti‐seizure medication (ASM) achieves seizure freedom in only about two‐thirds of patients. Drug resistance is encountered frequently in genetic epilepsies. Drug resistance affects particularly the clinically and genetically heterogeneous group of developmental and epileptic encephalopathies (DEE), the most severe forms of epilepsy.^1^ Since DEE are individually rare neurodevelopmental disorders, gene‐based knowledge on tailored precision medicine is limited. This is complicated even further as different variants in a given gene can have different effects such as loss of function (LoF) or gain‐of‐function (GoF) that can also react differently to specific ASM.^2^ In silico tools that allow for a reliable prediction of a variant effect, however, do not exist for most genetic epilepsies.^3^ Patients and their families are, thus, faced by repeated drug testing that requires successive slow introduction and, if unsuccessful, tapering off of multiple ASM. During this time, precious developmental avenues can pass by. This situation highlights the medical need for targeted treatment approaches including drug repurposing to rapidly and effectively reduce seizure load and comorbidities such as developmental delay/regression.

Traditional preclinical models, such as rodents or human‐induced pluripotent stem cells, are often time‐intensive, costly, and lack rapid applicability for personalized approaches. Alternative in vivo vertebrate models, such as Zebrafish, offer conserved neural circuitry and have been successfully used for disease modeling, drug discovery, and pharmacological testing.^4^ Despite whole‐genome duplication affecting a minority of genes, *SCN1Lab* and *SCN1Laa* models have recapitulated key DS phenotypes and responded to human‐relevant ASM.^5^ Their use at early developmental stages, before the onset of vertebrate‐specific regulation, allows for high‐throughput drug screening in many jurisdictions.^6^ However, their status as vertebrates also subjects their use to stricter country‐specific ethic regulations.^7^

*Drosophila melanogaster* or the vinegar fly has been applied as a powerful tool for human disease modeling for several decades, sharing approximately 65% of disease‐related human genes and having a relatively simple neuronal system, making it an ideal model for studying the effects of drugs and their therapeutic effects.^8^, ^9^ It enables precise, efficient, and rapid genetic manipulations. The vinegar fly has historically been used to study epilepsy since the discovery of bang‐sensitive mutants in 1973, which exhibit seizure‐like behavior and paralysis in response to mechanical shock.^10^ Other *Drosophila* mutants exhibiting seizures were subsequently identified with variants in Shaker (*sh*) and ether‐a‐go‐go (*eag*). The vertebrate homologs (*KCNQ*) of these genes were later found to be associated with epilepsy.^9^ In recent years, the vinegar or fruit fly has increasingly been used to study novel genetic causes of DEE and epilepsies, leveraging the versatile genetic toolbox it offers.^11^, ^12^

Here, we used *Drosophila melanogaster* to analyze its appropriateness as a rapid in vivo model for preclinical ASM testing in genetic epilepsies. For that, we have chosen Dravet syndrome (DS) as one of the most common DEE. DS is caused by variants in the *SCN1A* gene encoding for the sodium channel Nav1.1.^13^ Patients with DS show cognitive and motor deterioration in the course of the disease and hold a high rate of early mortality.^13^, ^14^ Several models of DS have been created in *Drosophila*, sharing the same variant in their ortholog gene (*para*).^15^, ^16^, ^17^ The *Drosophila melanogaster* models with *SCN1A* mutations by Sun et al. and Schutte et al. were created using ends‐out homologous recombination, enabling the precise insertion of human mutations into the para sodium channel gene, a homolog of *SCN1A*. Two clinically relevant mutations were modeled: (i) S1231R, associated with DS, and (ii) K1270T, linked to genetic epilepsy with febrile seizures plus (GEFS+) in humans.^15^, ^16^ Both mutations were introduced at homologous sites in the para gene to replicate their pathogenic effects. These models were reported to exhibit characteristic disease features, including reduced lifespan, increased susceptibility to spontaneous and heat‐induced seizures, and electrophysiological changes.^15^, ^16^

We propose that using *Drosophila* as a rapid preclinical in vivo model to test ASM efficacy can be used for genetic epilepsies.

## 2 METHODS

### 2.1 Fly stocks

Flies were kept on standard cornmeal at 25°C in a 12/12 h light/dark cycle using the following lines: DS (S1231R), GEFS+ (K1270T), which were kindly provided by Diane O'Dowd,^15^, ^16^ and w1118 (#3605) as wildtype controls from the Bloomington Drosophila Stock Center, Indiana University Bloomington, IN, USA (https://bdsc.indiana.edu).

### 2.2 Heat shock assay

To induce seizures, female flies aged 5–6 days were anesthetized using CO_2_ and placed in groups of five within transparent vials. After a 30‐min recovery period, we induced seizures by immersing vials containing the flies in a 40°C transparent water bath for 2 min. Seizure activity was recorded using the camera of an iPad Pro (10,5, 1st Generation) and analyzed at a later time point. Seizures were manually counted and defined as the inability to maintain an upright position, combined with twitching of the legs, wings, or full‐body contractions. The status of each fly was assessed every 10 s. Data from multiple experiments were used to calculate the average probability of seizure for a fly of a certain genotype every 10 s during the assay. Given that the *para* gene is localized on the X chromosome and heterozygous female flies exhibit increased variability in response to heat‐induced seizures, we exclusively utilized homozygous female flies, consistent with the approach outlined by Schutte et al.^16^

### 2.3 Anti‐seizure medication administration

To test the efficacy of ASM, 2–3‐day‐old female flies were transferred to vials containing the drug concentration as required and tested as described. The flies were transferred to food vials containing medication and kept on this diet for a duration of 3 days. The medication was carefully mixed into 1 g of standard cornmeal food. Clobazam (CLB, Frisium®, 10 mg tablets, Sanofi) was shredded and mixed directly into the food. Fenfluramine (FEN, Fintepla®, UCB) was directly pipetted and mixed into the food. Stiripentol (STP, Merck, Catalog number S6826), phenytoin (Merck, Catalog Number P1290000), and valproic acid (VPA, Selleck, Catalog No. S3944) were dissolved in DMSO (Merck, Catalog No. D2650) and then pipetted and added to the food. Controls for DMSO were conducted simultaneously if appropriate for drugs dissolved in DMSO.

### 2.4 Life span assay

Female flies emerging from a 24‐h cohort were selected and randomly put into different vials containing either standard food or standard food mixed with medication. Each group consisted of a minimum of 50 female flies. Fresh food was provided three times per week, and at the same time, the survival was checked.

### 2.5 Statistics

For comparison of treatment effects, a two‐way repeated measures analysis of variance (ANOVA) was used to compare rates of flies seizing. For comparison of mean seizure times, an ANOVA was used with a Tukey post‐hoc test. DMSO had no effect on seizures in both lines (DS: *F*[5,51] = 0.390, *p* = 0.853) (GEFS+: *F*(5,58) = 0.949, *p* = 0.457). We used the blank control as the baseline for statistical testing of flies where drugs were directly pipetted into the food (CLB and FEN) and the highest DMSO concentration for flies where drugs were dissolved in DMSO (STP, phenytoin, and VPA). *p* values < 0.05 were considered significant.

## 3 RESULTS

### 3.1 Survival and seizures

Control flies exhibited a maximum lifespan of approximately 75 days, whereas DS and GEFS+ flies demonstrated reduced survival under standard conditions. Specifically, GEFS+ flies showed a median survival reduction of 10 days (Figure S1). DS and GEFS+ flies exhibited seizures after a few seconds when heated to/above 40°C, which resembles the phenotype of heat‐induced seizures observed in patients.^15^, ^16^

### 3.2 ASM indicated in DS

To investigate the efficacy of ASM in reducing seizures in flies carrying patient variants, we utilized flies with human variants knocked into the orthologous *Drosophila* gene *para* at corresponding positions.^15^, ^16^ To test whether standard drug therapy approaches for patients with DS would also have a positive effect in the fly model, we applied VPA, CLB, STP, and FEN.

ASM were first tested on w1118 flies as a negative control. VPA (*F*[4, 40] = 1.090, *p* = 0.375), STP (*F*[4,40] = 2.595, *p* = 0.051), and FEN (*F*[4520] = 0.130, *p* = 0.972) had no effect on seizures over time, while CLB (*F*[4,40] = 3.498, *p* = 0.015) slightly increased the seizure rate at the highest dose of 10 mM (Figure S2).

**FIGURE 1:**
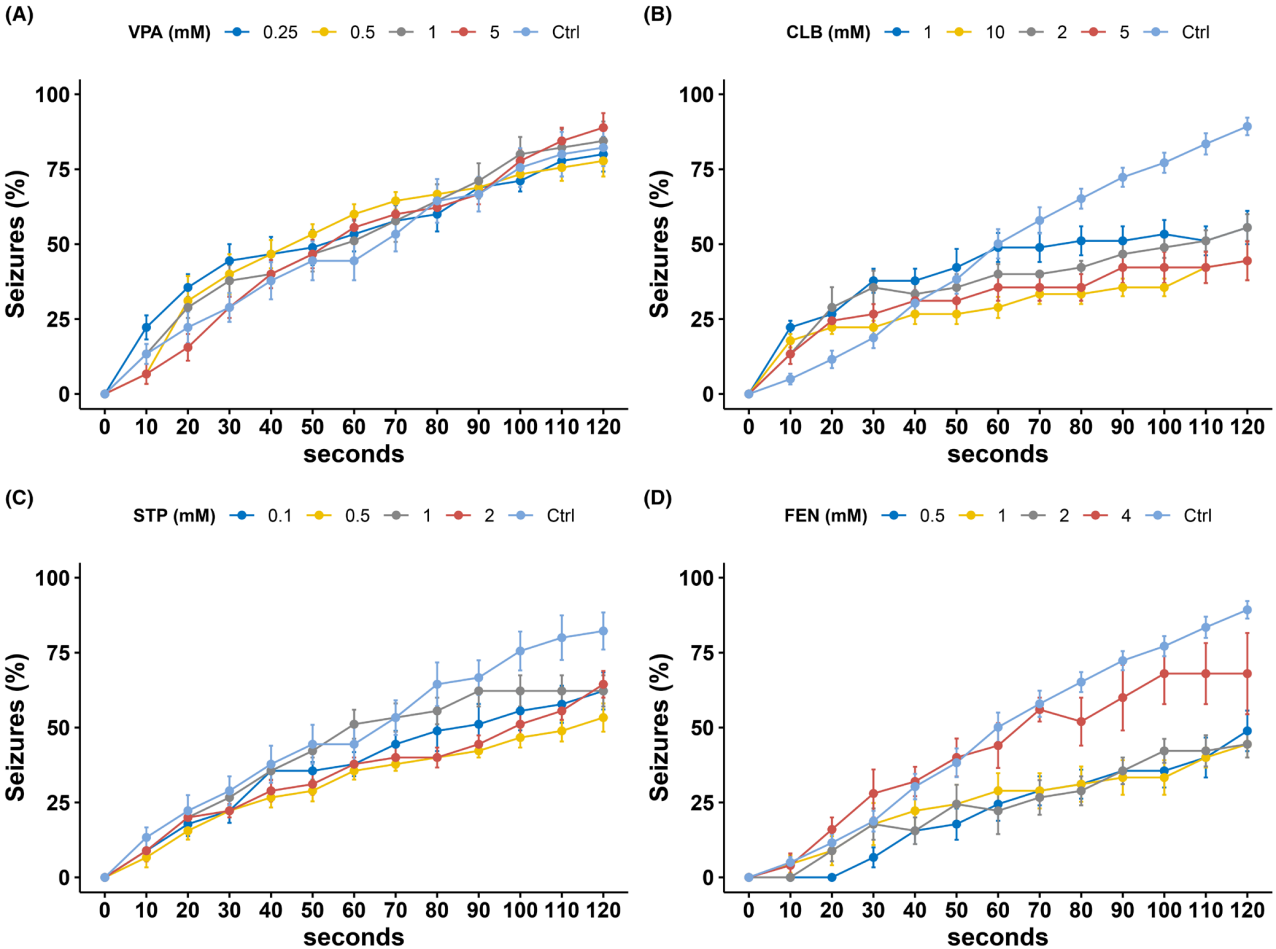
Seizure reduction through anti‐seizure medication in GEFS+ *Drosophila melanogaster*. (A) Valproic acid treatment did not reduce seizures significantly (*p* = 0.883), while (B) clobazam (*p* < 0.001), (C) stiripentol (*p* = 0.002), and (D) fenfluramine (*p* < 0.001) did. Ctrl, Control; CLB, clobazam; FEN, fenfluramine; GEFS+, generalized epilepsy febrile seizures+; STP, stiripentol; VPA, valproic acid. Data are presented as mean ± standard error.

VPA is the first‐line treatment for DS. Applying ASM in GEFS+ flies resulted in no significant seizure reduction (*F*[4,40] = 0.289, *p* = 0.883) (Figure 1A). However, treating the same model with CLB (*F*[4,55] = 7.890, *p* < 0.001) or STP (*F*[4,40] = 4.978 *p* = 0.002) both resulted in a significant reduction of seizures (Figure 1B,C). FEN, an ASM recently approved for the treatment of DS, significantly reduced seizures in GEFS+ flies in a dose‐dependent manner (*F*[4,36] = 14.448, *p* < 0.001) (Figure 1D). However, 4 mmol FEN appeared to have a smaller effect than lower doses, possibly due to toxicity. When quantifying mean seizure time, a dose‐dependent reduction in seizures was observed for CLB (*F*[4,55] = 7.928, *p* < 0.0001) (Figure S3B). Higher doses of FEN (*F*[4,55] = 14.449, *p* < 0.0001) and STP (*F*[4,40] = 5.251, *p* = 0.002) did not result in greater seizure reduction (Figure S3C,D), while VPA did not reduce seizure time (*F*[4,40] = 0.262, *p* = 0.9) (Figure S3A).

**FIGURE 2:**
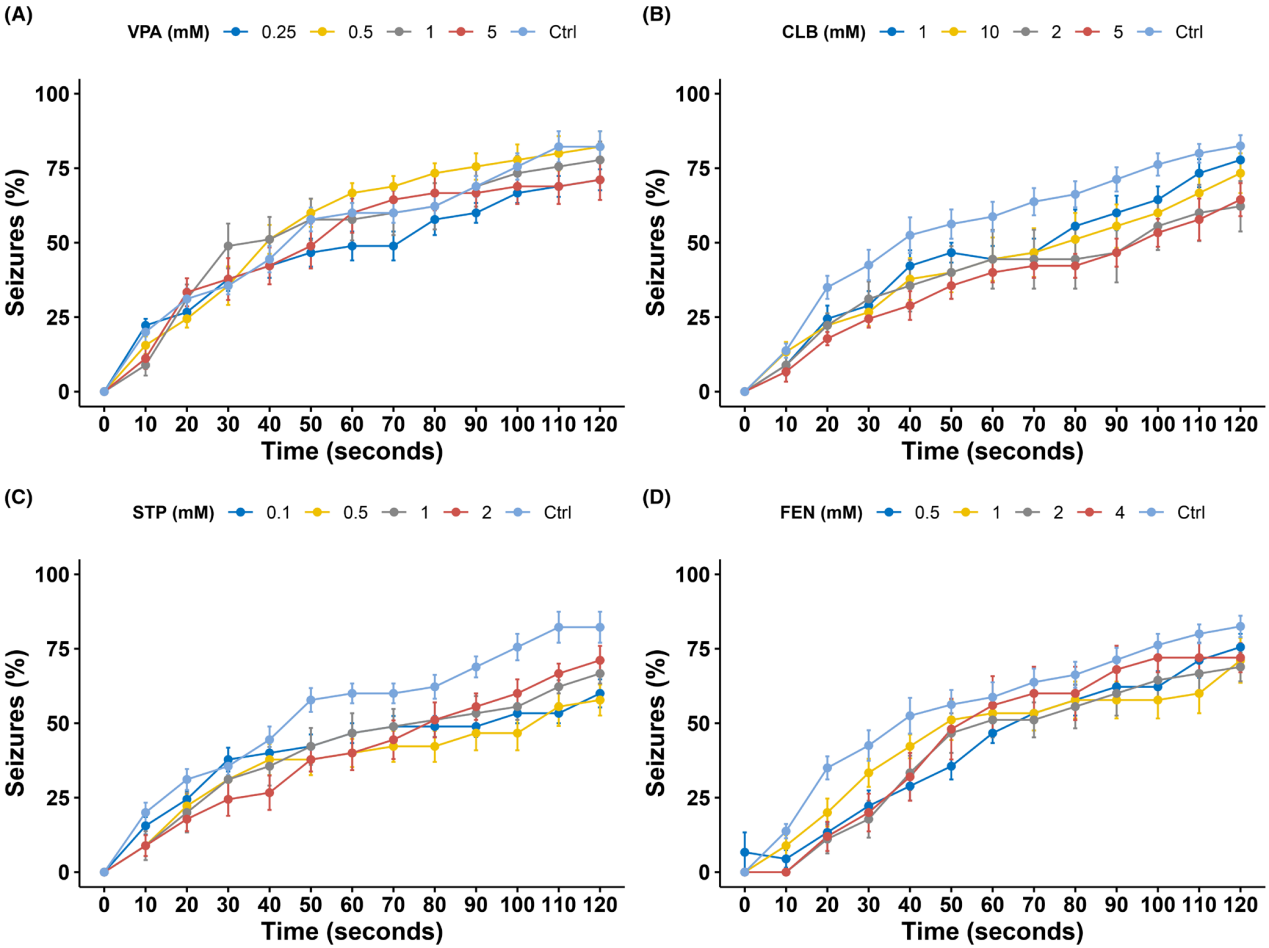
Seizure reduction through anti‐seizure medication in DS *Drosophila melanogaster*. (A) Valproic acid treatment did not reduce seizures significantly (*p* = 0.515), as well as fenfluramine (*p* = 0.068) (D). Clobazam (*p* = 0.017) (B), and stiripentol reduced seizures significantly (*p* < 0.014) (C). Ctrl, Control; CLB, clobazam; FEN, fenfluramine; GEFS+, generalized epilepsy febrile seizures+; VPA, valproic acid; STP, stiripentol. Data are presented as mean ± standard error.

In a *Drosophila* model with variants associated with DS, testing of the same drugs revealed that VPA (*F*[4,40] = 0.828, *p* = 0.515) and FEN (*F*[4,43] = 2.360, *p* = 0.068) exhibited no significant seizure reduction during 120 seconds of heating, while CLB (*F*[4,47] = 3.366, *p* = 0.017) and STP (*F*[4,40] 3.583, *p* = .014) did (Figure 2). Quantifying mean seizure times, we found, however, that CLB (F[4,47] = 3.366, *p* = 0.017), STP (*F*[4,40] = 3.583, *p* = 0.014), and FEN (*F*[4,47] = 2.99, *p* = 0.028) did reduce mean seizure time, while VPA (*F*[4,40] = 0.828, *p* = 0.515) did not (Figure S4).

In the next step, we tested whether using standard ASM as a long‐term treatment under standard conditions would rescue the decreased survival. Here, we found that the administration of FEN in GEFS+ and DS flies increased survival in both lines (Figure S1).

### 3.3 ASM contraindicated in DS

Sodium channel blockers are generally believed to be contraindicated in DS, especially as a long‐term treatment, as they can increase seizures and can lead to long‐term negative effects on neurocognitive outcomes.^14^, ^18^ Therefore, we sought to determine whether a similar effect would be observed in *Drosophila*. Our findings indicate that while phenytoin increased seizures in GEFS+ flies, higher dosages were required to elicit this effect in DS flies (Figure S4A,B).

## 4 DISCUSSION

Here, we report the utilization of the fruit fly, *Drosophila melanogaster*, as a rapid in vivo tool for the screening of ASM in genetic epilepsies, employing flies with variants in their ortholog gene. Our results demonstrate that several ASMs known to be clinically effective in DS reduce seizure burden in these models, with differential responses observed between the two lines carrying the S1231R and K1270T variants, respectively. VPA was found to be less effective in this context. In other *Drosophila melanogaster* models of epilepsy, such as bang‐sensitive flies, treatment with VPA resulted in a reduction in seizure activity.^19^, ^20^ However, in those studies, VPA was directly administered either through injection into the circulatory system or into the head.^19^, ^20^ The observed lack of seizure reduction in the VPA treatment group in our study may be attributed to the flies' detoxification systems that are bypassed by direct administration. Further investigation is required to elucidate this phenomenon.

Differences in ASM efficacy were observed between the two models GEFS+ and DS, with greater efficacy in the GEFS+ model, supporting the DS model's representation of intractable epilepsy. Our DS model exhibits a typical LoF variant, reducing sodium current magnitude and increasing the activation threshold.^16^ This results in decreased excitability in GABAergic neurons. At elevated temperatures, the mutation triggers heat‐induced seizures, resembling DS symptoms in humans. The GEFS+ model mutation is a GoF variant that lowers the threshold for sodium current activation and broadens the range of persistent sodium currents in *Drosophila*. Although in human *SCN1A*‐associated epilepsies, gain‐of‐function variants can result in congenital arthrogryposis, neonatal‐onset epilepsy, tonic seizures, and apnea, the K1270T mutation has been clinically linked to GEFS+.^2^ This disparity highlights limitations in our preclinical model and underscores the need for refined approaches.

We note that while these Drosophila lines carry human orthologous mutations associated with DS and GEFS+, they should not be interpreted as complete phenocopies of the respective syndromes but rather as functional systems for pharmacological screening in genetic epilepsies. While likely due to differences in experimental design, we did not observe the severe baseline phenotype of DS flies described in the original study; mean seizure time was similar. Nevertheless, the use of the fruit fly offers numerous advantages for studying genetic epilepsies, preclinical assessment of ASM, and the identification of novel treatments complementing *other* in vivo systems such as the zebrafish. While zebrafish as a vertebrate offer more similarities in neural architecture compared to mammals, *Drosophila* offers high levels of genetic tractability and throughput. Together, these models could form a complementary toolkit that enables translational research at multiple biological levels. These include the rapid availability of the fly, the low cost and maintenance requirements, fewer ethical restrictions, and the rapid and versatile genetic toolkits. Despite the significant differences in brain anatomy between flies and humans, the fundamental functions of neurons are largely conserved, making the fly an accessible and useful tool. The utilization of tools such as *UAS‐Gal4/Crimic*(*T2A‐GAL4*) permits the genetic manipulation of specific cell types, inducing a multitude of genetic variants and regulating expression.^8^, ^21^ Moreover, these tools permit the expression of human cDNA under control of endogenous promoters, thus enabling the assessment of variant effects, performing rescue experiments, or, as proposed here, studying the effect of different drugs on various behavioral phenotypes.^21^

## 5 CONCLUSION

*Drosophila melanogaster* models carrying mutations associated with human genetic epilepsies provide a rapid in vivo system that enables functional assessment of variant‐specific effects and preclinical evaluation of ASM efficacy. Although the fly will not be able to recapitulate the full complexity of human syndromes, it offers a cost‐effective and genetically flexible platform that can bridge mechanistic insight and therapeutic discovery. Future efforts applying this system to broader variant spectra and additional gene targets may accelerate the development of tailored treatments in genetic epilepsies.

## AUTHOR CONTRIBUTIONS

KLM, DO, and AMK contributed to the conception and design of the study. EVT, MMH, and KLM conducted experiments and performed the data analysis. All the authors discussed the results, revised the first draft, and contributed to the final manuscript.

## CONFLICT OF INTEREST STATEMENT

KLM and AMK received honoraria for lecturing and travel expenses from Angelini Pharma and Jazz Pharmaceuticals. AMK is an advisory board member of Desitin and received honoraria for a manuscript from Neuraxpharm. The remaining authors have no conflict of interest. We confirm that we have read the Journal's position on issues involved in ethical publication and affirm that this report is consistent with those guidelines.

## Supporting information


Data S1.

## Data Availability

The data that support the findings of this study are available from the corresponding author upon reasonable request.
