# Supplementary material for: Drosophila melanogaster as a rapid in vivo assay system for preclinical anti‐seizure medication testing
Source: Epilepsia Open. 2025 Jul 10;10(4):1260–7. doi: 10.1002/epi4.70101 (PMC12362156; doi:10.1002/epi4.70101)
Supplement: Supplementary file 1 — Data S1. [file EPI4-10-1260-s001.docx]

**Supplemental Figures**

**Supplemental Figure 1. Survival of DS and GEFS+ flies treated with fenfluramine.** (A) GEFS+, (B) and DS flies exhibit reduced survival that is rescued by administration of fenfluramine. Abbreviations: GEFS+, Generalized epilepsy febrile seizures+; DS, Dravet syndrome.

**Supplemental Figure 2. Seizure induction and anti-seizure medication testing in wildtype flies.** Abbreviations: VPA, valproic acid; CLB, clobazam; STP, stiripentol; FEN, fenfluramine. Data is presented as mean ± standard error.

**Supplemental Figure 3. Mean seizure reduction following treatment of GEFS+ flies with various ASM.** Seizure durations were measured across increasing concentrations of valproate (A), clobazam (B), stiripentol (C), and fenfluramine (D). Significant seizure reductions were observed for clobazam, stiripentol, and fenfluramine, but not for valproate. Data is presented as mean ± standard error. Abbreviations: Ctrl, Control;

**Supplemental Figure 4. Mean seizure reduction following treatment of DS flies with different ASM.** Seizure durations were measured across increasing concentrations of valproate (A), clobazam (B), stiripentol (C), and fenfluramine (D). Significant seizure reductions were observed for clobazam, stiripentol, and fenfluramine, but not for valproate. Data is presented as mean ± standard error.

**Supplemental Figure 5. Mean seizure reduction following treatment of (A) GEFS+ and (B) DS flies with phenytoin.** Data is presented as mean ± standard error.
